# Switchable Terahertz Metasurfaces for Spin-Selective Absorption and Anomalous Reflection Based on Vanadium Dioxide

**DOI:** 10.3390/s24144548

**Published:** 2024-07-13

**Authors:** Jinxian Mao, Fengyuan Yang, Qian Wang, Yuzi Chen, Nan Wang

**Affiliations:** 1 School of Microelectronics, Shanghai University, Shanghai 200444, China; mjx13621805747@shu.edu.cn (J.M.); wangqianyi@shu.edu.cn (Q.W.); chen_yuzi@shu.edu.cn (Y.C.); 2 Shanghai Collaborative Innovation Center of Intelligent Sensing Chip Technology, Shanghai University, Shanghai 200444, China

**Keywords:** chiral metasurface, switchable, terahertz, spin-selective, Pancharatnam–Berry phase

## Abstract

Conventional chiral metasurfaces are constrained by predetermined functionalities and have limited versatility. To address these constraints, we propose a novel chirality-switchable terahertz (THz) metasurface with integrated heating control circuits tailored for spin-selective anomalous reflection, leveraging the phase-change material vanadium dioxide (VO_2_). The reversible and abrupt insulator-to-metal phase transition feature of VO_2_ is exploited to facilitate a chiral meta-atom with spin-selectivity capabilities. By employing the Pancharatnam–Berry phase principle, complete 2π reflection phase coverage is achieved by adjusting the orientation of the chiral structure. At the resonant frequency of 0.137 THz, the designed metasurface achieves selective absorption of a circularly polarized wave corresponding to the state of the VO_2_ patches. Concurrently, it reflects the circularly polarized wave of the opposite chirality anomalously at an angle of 28.4° while maintaining its handedness. This chirality-switchable THz metasurface exhibits promising potential across various applications, including wireless communication data capacity enlargement, polarization modulation, and chirality detection.

## 1. Introduction

Chirality, referring to asymmetry of an object that prevents it from aligning with its mirror image, is a captivating phenomenon observed in nature. Numerous organic molecules, including sugars, amino acids, and pharmaceutical compounds, possess chiral structures. These structures cause them to interact differently with left-handed circularly polarized (LCP) and right-handed circularly polarized (RCP) waves, leading to circular dichroism (CD) [1] and optical activity effects [2]. LCP and RCP are defined distinctively as follows. When observed along the direction of propagation, the RCP wave is characterized by an electric field vector rotating clockwise, whereas the LCP wave exhibits an electric field vector rotating counterclockwise. Such resonance response is usually observed within the terahertz (THz) spectrum. The integration of THz-scale technology with chirality holds immense promise for applications in biomolecular sensing [3,4], non-destructive testing [5], and sixth-generation (6G) wireless communication [6,7]. Polarization, alongside attributes of amplitude, phase, and frequency, provides another degree of freedom for electromagnetic wave modulation in 6G THz wireless communication. Moreover, its handedness-sensitive nature bolsters the security of THz communication. Because of the typically subtle chiral characteristics of natural molecules, various chiral metamaterials have been proposed to enhance the chirality response at THz [8]. The chiral metasurface is a two-dimensional subwavelength device made up of periodic electromagnetic meta-atoms, which can modulate the amplitude [9,10], phase [11,12,13], and polarization [14,15,16,17,18,19,20,21,22] of the incident electromagnetic waves.

Recently, there have been advancements in chiral metasurfaces tailored for spin-selective reflection, enabling the reflection of a specific circularly polarized (CP) wave while absorbing the other one [23,24,25,26,27,28,29,30,31]. For instance, Tang et al. provided both theoretical and experimental demonstrations of a chiral metasurface with η-shaped metallic resonators on top of a dielectric spacer. This chiral metasurface achieved a maximum absorption of over 80% and a CD value of approximately 0.5 in the visible frequencies [30]. Qureshi et al. designed an ultra-thin chiral metasurface incorporating a mushroom-shaped resonator, which attained a peak absorption rate of 98.49% for incident LCP waves and only 7.39% for RCP waves at 12.4 THz, resulting in a remarkable CD value of 91.1% [31]. Nevertheless, once these chiral metasurfaces are fabricated their properties tend to be fixed, greatly limiting their functionality and efficiency. To address this challenge, the integration of tunable materials into chiral metasurfaces undeniably presents an optimal solution. Among these materials, vanadium dioxide (VO_2_), a phase change material, boasts advantages such as a phase change temperature close to room temperature and a notable contrast in performance before and after the phase transition [32]. A number of VO_2_-based tunable chiral metasurfaces operating in the THz and GHz bands have been proposed [33,34,35,36,37,38,39]. Wang et al. introduced a chiral metasurface comprising a C-shaped metal open-end ring and an L-shaped VO_2_ configuration. This innovative design enables seamless switching of the CD response between the conduction and cut-off states while maintaining a CD value exceeding 0.9 within the frequency range of 5.70 to 8.55 THz [37]. However, despite their thermal regulation capabilities, these VO_2_ metasurfaces are limited to a single function of chirality-selective absorption, and lack compatibility with electronic devices.

**Figure 1:**
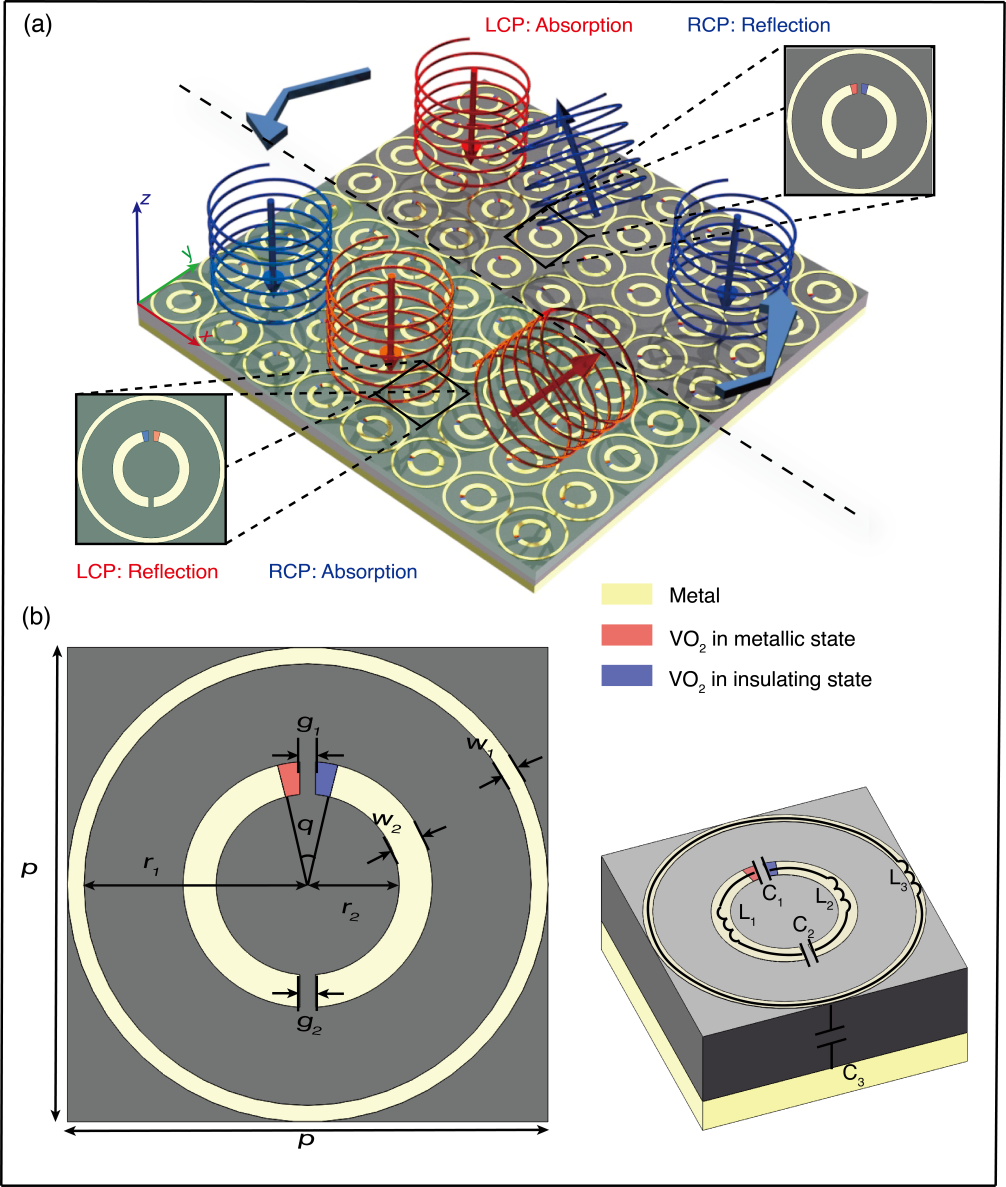
(**a**) An illustration depicting the chirality-switchable THz metasurface designed for spin-selective absorption and anomalous reflection. The enlarged meta-atom shows the metallic structure embedded with VO_2_ patches. By selectively heating the VO_2_ patch in the metallic or insulating states, the metasurface is capable of deflecting a particular CP wave without altering its handedness while efficiently absorbing the other CP wave. (**b**) Schematic illustrations of the meta-atom: (**left**) top view with dimension parameters and (**right**) perspective view with an equivalent labeled circuit of the meta-atom.

In this paper, we propose a spin-selective switchable chiral metasurface with integrated heating control circuits. The multifunctional metasurface is designed with a metallic split-ring resonator (SRR) integrated with VO_2_ patches. Driving a direct current bias current to the heating control circuit and leveraging the reversible and abrupt insulator-to-metal phase transition characteristics of VO_2_ allows for the reconstruction of the SRR’s geometry, leading to a transition in CD values [40] from −0.938 to 0.938. This design enables dynamic control of both spin-selective absorption and anomalous reflection for CP THz waves, as illustrated in Figure 1a. According to the Pancharatnam–Berry (PB) phase principle, an entire 2π phase modulation is realized by adjusting the orientation of the meta-atoms. As a proof-of-concept demonstration, a gradient chiral metasurface for spin-selective anomalous reflection is designed by tailoring the spatial distribution of the meta-atoms. When the VO_2_ patches are in the insulating or metallic state, the metasurface does not demonstrate chiral properties, deflecting both LCP and RCP waves equally; however, upon heating a selected VO_2_ patch to the metallic state, the metasurface acquires chiral properties, resulting in a significant CD value (0.938) at the resonant frequency of 0.137 THz. Consequently, at this resonant frequency the chiral metasurface selectively absorbs LCP waves while exhibiting anomalous co-polarized reflection of RCP waves. Within the non-resonant frequency range of 0.13 THz–0.15 THz, the metasurface exhibits equal reflectivity for both the LCP and RCP waves.

## 2. Metasurface Design and Demonstrations

### 2.1. Meta-Atom Design

Figure 1b depicts the schematic structure of the chiral meta-atom, consisting of a sandwich-like metal–insulator–metal (MIM) configuration. The dielectric spacer is constructed from sapphire, with a thickness of 180 μm and a relative permittivity of 9.61. A metallic layer is placed at the bottom for enhanced reflection and zero transmission. The top layer consists of an outer metallic ring and an inner SRR. The outer metallic ring has an inner radius $r_{1}$ of 282.5 μm and a width $w_{1}$ of 5 μm. The inner SRR, comprised of metal-integrated VO_2_ patches, has an inner radius $r_{2}$ of 110 μm, a width $w_{2}$ of 40 μm, a gap width $g_{1}$ of 19 μm, and a top VO_2_ angle *q* of 28°. To study the electromagnetic response of the meta-atom, we analyzed its structure with the equivalent circuit theory. Within the inner SRR, the two gaps represent the inner equivalent capacitance C_1_ and C_2_ coupled with the inductance from the metallic arc L_1_ and L_2_, leading to a resonant frequency of 0.137 THz. The space between the inner SRR and the outer metal ring introduces an additional outer capacitance C_3_, yielding a second resonant frequency of 0.16 THz. The MIM structure itself can also be considered as an equivalent capacitance C_4_, which is affected by the shape of the upper Al/VO_2_. Altering the heating state of the inner SRR VO_2_ patch effectively adjusts the inner equivalent capacitance without affecting the outer equivalent capacitance. This alteration directly influences the circuit properties, leading to a significant change in the electromagnetic response to CP waves at the targeted resonant frequency of 0.137 THz, whereas the electromagnetic response remains unaffected at the redundant frequency of 0.16 THz.

**Figure 2:**
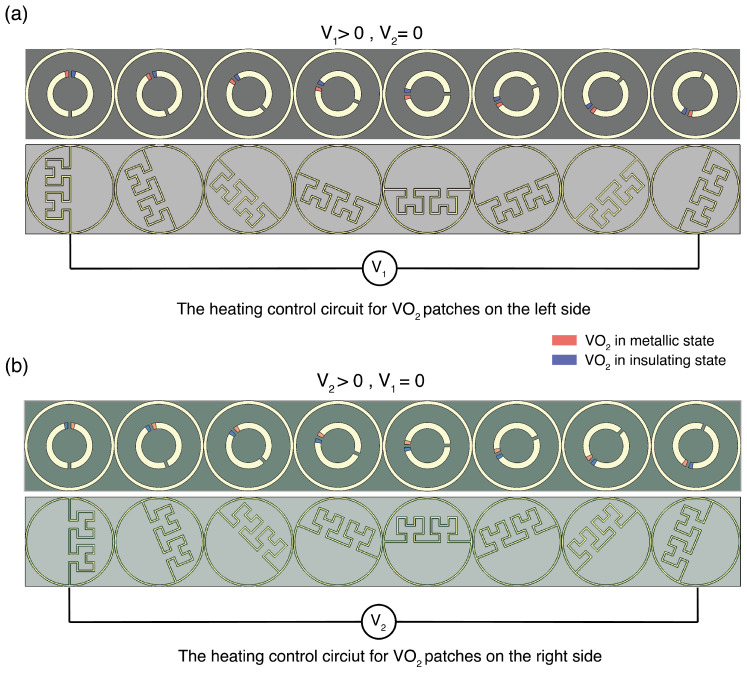
The proposed two-layer heating control circuit for selective control of the thermal states of VO_2_ patches at the meta-atom array level. This circuit is specifically tailored for the VO_2_ patches located on (**a**) the left side and (**b**) the right side of the SRR.

The potential heating control circuits in the meta-atom array level are presented in Figure 2. Two temperature control circuits, each incorporating Peano-shaped microheaters at different configurations, are separately situated in two layers underneath the VO_2_ patches [41,42]. The Peano-shaped microheater employs mathematical fractal theory to strategically position high-density resistive wires on either the left or right side of the meta-atom. This arrangement produces substantial heat due to the resistance encountered by the electric current flowing through the metal wire. This layout ensures that precise heating is directed to the specified zones. Upon activation of the microheater, heat is generated and efficiently conveyed to the upper layer of the patterned meta-atom structure, leading to an elevation in the temperature within the designated VO_2_ patches. By biasing a current through the control circuit in the corresponding layer, the VO_2_ patches on either the left or right side of the SRR can be selectively heated as needed. Upon applying bias voltage V_1_, as depicted in Figure 2a, the VO_2_ patches located on the left side of the meta-atoms experience thermal activation, enabling the meta-atoms to absorb the LCP wave and reflect the RCP wave at the resonant frequency. Similarly, in Figure 2b, when a bias voltage V_2_ is applied, the VO_2_ patches that are situated on the right side of the meta-atoms become thermally activated, providing the meta-atoms with the capability to reflect the LCP wave and absorb the RCP wave at the resonant frequency. At room temperature (≈298 K), VO_2_ functions as an insulator with an electrical conductivity of $\sigma_{0}$ ≈ 140 S m^−1^. When heated above its phase transition temperature (≈341 K), VO_2_ undergoes a reversible change from a low-temperature insulator phase to a high-temperature metal phase, increasing its conductivity up to 500,000 S $m^{-1}$. The meta-atom has a periodicity *p* of 575 μm. All metal used consisted of aluminium (Al), possessing a conductivity σ of 3.7 × $10^{7}$ S $m^{-1}$ [43]. The meta-atom exhibits diverse responses to electromagnetic waves under different heating conditions, resulting in distinct regulatory effects on circularly polarized (CP) waves across the entire metasurface.

The metal–insulator transitions (MIT) of VO_2_ influence the electromagnetic response of meta-atoms to CP waves. Despite the nanosecond-scale MIT of VO_2_, in most instances the switching speed of a device is constrained primarily by external elements, including device dimensions, parasitic resistance and capacitance, and the characteristics of VO_2_, rather than its inherent switching capabilities. In this paper, the switching time is related to the volume of VO_2_ that requires heating. Transitions with rise times of 2 ns have been reported [44] when VO_2_ is grown directly on metal electrode tips spaced a few hundred nanometers apart. We anticipate utilizing Joule heating to achieve rapid actuation speeds within 40 μs [45].

Employing beam deflection for directional transmission effectively counteracts the path loss experienced by shorter wavelengths during THz wave transmission. Concurrently, spin-selective reflection enhances the anti-interference performance of wireless THz wave transmission. Both factors contribute to the advancement of terahertz-scale communication technology. To realize spin-selective anomalous reflection, it is crucial to consider the properties of spin-selective absorption, handedness preservation, and full phase coverage in the metasurface meta-atom design. To implement the first two functions, we utilized transmission matrix theory to analyze the structural characteristics of the meta-atoms. We applied the Jones matrix to establish the relationship between the incident and reflected electric fields of the reflective metasurface [46]. The incident electric field $E_{in}$ and reflected electric field $E_{re}$ can be mathematically expressed as follows:

(1) $$\left(\begin{array}{c} E_{re}^{x}\\ E_{re}^{y} \end{array}\right)=\left(\begin{array}{cc} r_{xx} & r_{xy}\\ r_{yx} & r_{yy} \end{array}\right)\left(\begin{array}{c} E_{in}^{x}\\ E_{in}^{y} \end{array}\right)=R\left(\begin{array}{c} E_{in}^{x}\\ E_{in}^{y} \end{array}\right).$$

In the above, the linear reflection coefficients are represented as $r_{xx}$, $r_{xy}$, $r_{yx}$, and $r_{yy}$, with both incident and reflected fields polarized along either the *x*- or *y*-axis, while R is the reflection or transmission matrix of the metasurface in a Cartesian coordinate system. The Jones vectors $\sqrt{2}{\left(1,-i\right)}^{⊤}/2$ and $\sqrt{2}{\left(1,i\right)}^{⊤}/2$ represent the LCP and RCP waves, respectively. The reflection matrix R in the linear polarization basis can be converted into the reflection matrix $R_{cir}$ in the circular polarization basis as follows:

(2) $$\begin{array}{cc} R_{\mathrm{cir}} & \left.=\left(\begin{array}{cc} r_{LR} & r_{LL}\\ r_{RR} & r_{RL} \end{array}\right.\right)\\ \; & =P^{-1}RP\\ \; & =\frac{1}{2}\left(\begin{array}{cc} r_{xx}+r_{yy}+i\left(r_{xy}-r_{yx}\right) & r_{xx}-r_{yy}-i\left(r_{xy}+r_{yx}\right)\\ r_{xx}-r_{yy}+i\left(r_{xy}+r_{yx}\right) & r_{xx}+r_{yy}-i\left(r_{xy}-r_{yx}\right) \end{array}\right) \end{array}$$

where P is defined as $\frac{1}{\sqrt{2}}\left(\begin{array}{cc} 1 & 1\\ i & -i \end{array}\right)$. The subscripts “*R*” and “*L*” respectively represent the RCP and LCP waves. In this paper, we focus on the scenario of spin-selective anomalous reflection, where the metasurface absorbs the LCP wave while completely reflecting the RCP wave without altering its handedness. We establish the following conditions: $r_{L}L=r_{R}L=r_{L}R$ = 0, and $r_{R}R$ = 1. By employing Equations (1) and (2), the reflection matrix can be derived from this particular case as follows:

(3) $$R=\left(\begin{array}{cc} r_{xx} & r_{xy}\\ r_{yx} & r_{yy} \end{array}\right)=-\frac{e^{i\varphi }}{2}\left(\begin{array}{cc} -1 & i\\ i & 1 \end{array}\right)$$

where φ represents an arbitrary phase shift introduced by the chiral metasurface. The eigenvalues of the reflection matrix R being zero, along with the eigenvector ${\left(1,-i\right)}^{⊤}$, indicates that when a linearly polarized (LP) beam interacts with the metasurface, the LCP component of the LP wave is absorbed and the RCP component is completely reflected.

Table 1 provides an overview of the metasurface’s functionalities concerning CP waves across four different heating conditions. Regardless of the VO_2_ heating state, the metasurfaces consistently exhibit anomalous reflection of CP waves at non-resonant frequencies. However, the behavior of the metasurface at its targeted resonant frequency depends on the heating state of the VO_2_ patches. Specifically, when both VO_2_ patches are in either the insulated or metallic state, the symmetric meta-atom structure reflects both LCP and RCP waves. In the case where both VO_2_ patches are insulated, the amplitude is halved compared to non-resonant frequencies due to its resonant plasmonic structure. Furthermore, due to the asymmetric structure, the metasurface selectively absorbs either left-handed or right-handed CP waves based on the heated status of the VO_2_ patches.

By selectively heating the VO_2_ patches to their metallic state, the symmetry of the meta-atom structure is disrupted, transforming the meta-atom into a chiral configuration. Consequently, this alteration leads to different reactions to CP waves based on their handedness, thereby inducing circular dichroism. Circular dichroism refers to the distinct absorption of the LCP or RCP wave. Without considering higher-order diffraction, the absorption of LCP and RCP waves, defined respectively as $A_{LCP}$ and $A_{RCP}$, can be expressed as follows.

(4) $$A_{LCP}=1-{\left|r_{LL}\right|}^{2}-{\left|r_{LR}\right|}^{2}$$

(5) $$A_{RCP}=1-{\left|r_{RR}\right|}^{2}-{\left|r_{RL}\right|}^{2}$$

The reflection coefficient is denoted by $r_{ij}$; the subscript *i* indicates the polarization of the reflected field, while the subscript *j* corresponds to the polarization of the incident field. In addition, $r_{L}L$ and $r_{R}R$ represent the co-polarization reflection coefficients of the elements, while $r_{R}L$ and $r_{L}R$ represent the cross-polarization reflection coefficients. As observed along the wave propagation direction, the subscripts “*L*” and “*R*” indicate respectively the LCP and RCP waves. Given that the reflected electromagnetic wave transmits in the opposite direction to the incident wave, the cross-polarization reflected electric field rotates in the same direction as the incident electric field, whereas the co-polarization reflected electric field rotates in the opposite direction to the incident electric field. For instance, consider an RCP incident wave that propagates along the +z direction, characterized by its electric field vector rotating in a clockwise manner around the +z direction. The LCP and RCP waves propagating in the −z direction, resulting from the incident RCP wave, can be respectively categorized as cross-polarization and co-polarization reflection components. From the +z direction of incident wave propagation, the electric field vector of the cross-polarization reflected component LCP rotates clockwise, whereas the electric field vector of the co-polarized reflected component RCP rotates counterclockwise. Given that the reflected wave travels along the −z direction, its direction of rotation undergoes reversal.

**Figure 3:**
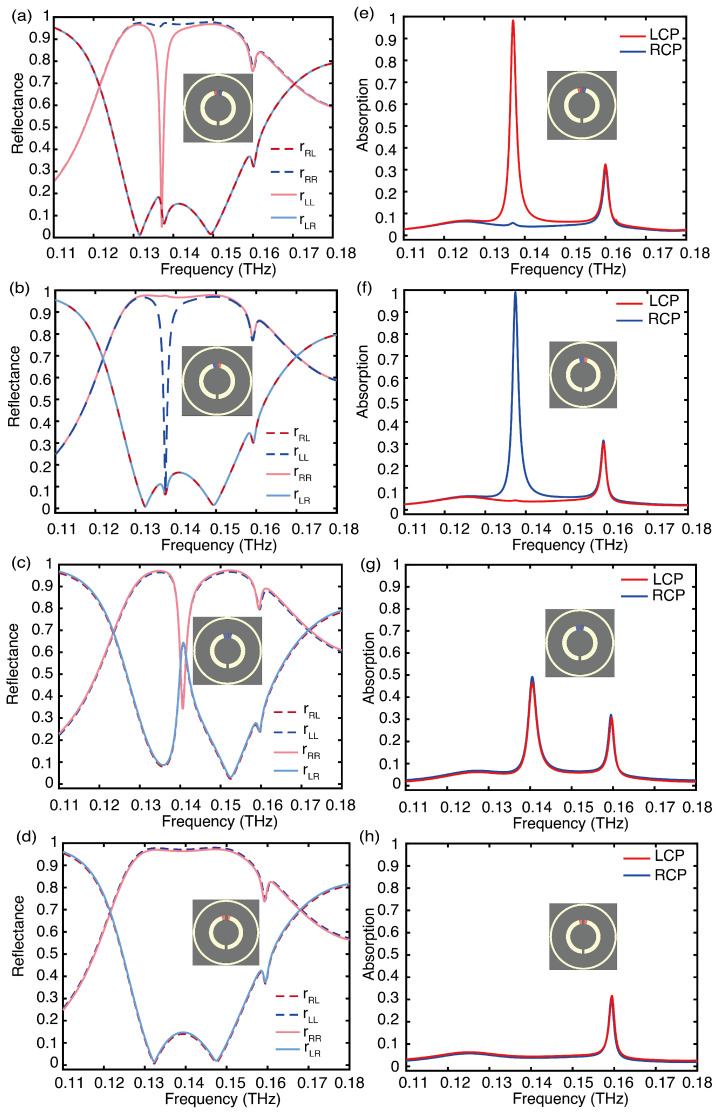
Characterization of the meta-atom for VO_2_ patches under different heating conditions: (**a**–**d**) show the simulated reflectance and (**e**–**h**) the absorption spectra of the meta-atom with different CP waves at normal incidence.

Due to the symmetric behavior of the meta-atom, we utilize the VO_2_ heating state depicted in Figure 1b as an example. The corresponding simulated reflection and absorption spectra are presented in Figure 3a,e, showcasing the electromagnetic reaction of the meta-atom to CP waves. At the non-resonant frequency, the meta-atom demonstrates cross-polarization reflection ($r_{R}L$ and $r_{L}R$) below 20% and co-polarized reflection ($r_{R}R$ and $r_{L}L$) above 90% within the frequency range of 0.13 THz to 0.15 THz. Moreover, due to its resonant plasmonic structures, the co-polarized reflection $r_{LL}$ approaches zero at the resonant frequency of 0.137 THz, where the LCP is absorbed rather than deflected and the RCP is fully reflected within the frequency range of 0.13 THz to 0.15 THz, as shown in the absorption spectra in Figure 3e. The other opposite heating state in Figure 3b,f produces a similar electromagnetic response, but reverses the handedness of the absorbed and reflected waves.

The simulated reflection and absorption spectra for the two VO_2_ patches in the insulated and metallic states are shown in Figure 3c,d, respectively. In both heating conditions, the structure is achiral, resulting in equal absorption of LCP and RCP. The simulated reflection differs only at the resonant frequency, where it is shifted to 0.14 THz due to structural changes. At non-resonant frequencies, the meta-atom exhibits cross-polarization reflection ($r_{R}L$ and $r_{L}R$) below 20% and co-polarized reflection ($r_{R}R$ and $r_{L}L$) above 90% within the frequency range of 0.13 THz to 0.15 THz. When the VO_2_ patches are in insulated states, the co-polarization reflection ($r_{R}R$ and $r_{L}L$) coefficient mutate to 0.63 and the cross-polarization reflection ($r_{R}L$ and $r_{L}R$) coefficient mutate to 0.36 at the resonant frequency. The absorption rates of RCP and LCP also mutate to 0.5, as shown in Figure 3g. This heating state can be considered a superposition of the heating states in Figure 3a,b. However, when the VO_2_ patches are in metallic states, there is no mutation, as depicted in Figure 3h. At non-resonant frequencies, both achiral structures reflect RCP and LCP within the frequency range of 0.13 THz to 0.15 THz.

**Figure 4:**
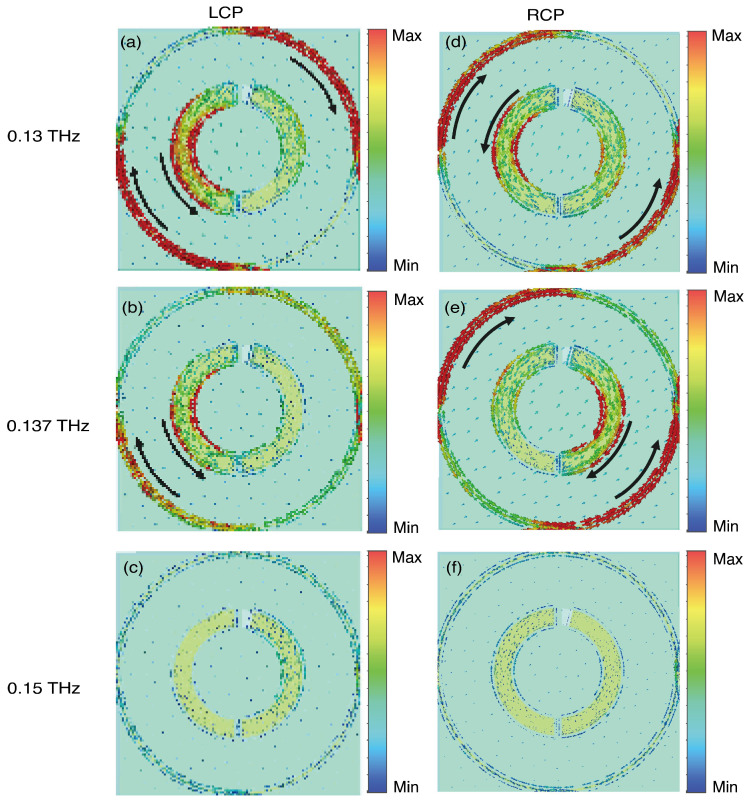
The surface current distributions of the meta-atom are induced when subjected to normally incident LCP waves at frequencies of (**a**) 0.13 THz, (**b**) 0.137 THz, and (**c**) 0.15 THz as well as to RCP waves at frequencies of (**d**) 0.13 THz, (**e**) 0.137 THz, and (**f**) 0.15 THz.

To delve more deeply into the physical mechanisms behind chiral absorption of meta-atoms, we utilized the VO_2_ heating state depicted in Figure 3a as a case study. Figure 4 shows the surface current distribution simulated at 0.13 THz, 0.137 THz, and 0.15 THz under normal incidence of LCP and RCP waves. In Figure 4a,d,e, the meta-atom exhibits three prominent currents. When opposing currents nullify each other, leaving only one current, it forms an electric dipole. These induced currents emit secondary radiation, facilitating high-efficiency reflection of the CP wave. Figure 4b indicates that at 0.137 THz the LCP wave excites a pair of counterparallel currents of nearly identical magnitude in both the outer metal ring and the inner SRR. These antiparallel currents act as two electric dipoles with a phase difference of π. Their radiated energies cancel each other out in the far-field region, resulting in high absorption of the LCP wave. Figure 4c,f depicts weak current excitation when CP waves illuminate the meta-atom at 0.15 THz, allowing for efficient reflection of the CP waves.

**Figure 5:**
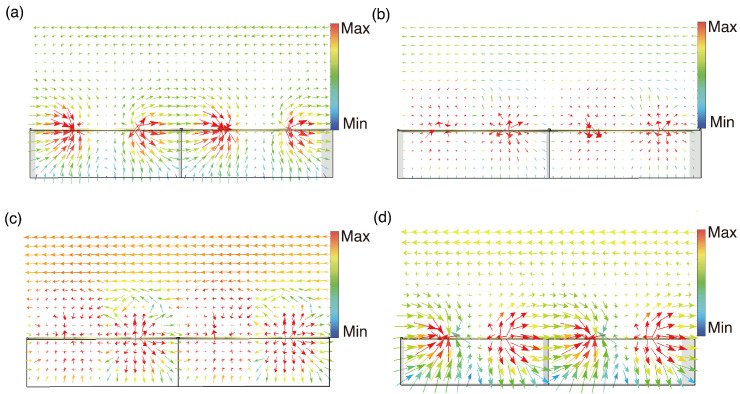
The electric field distribution of the chiral meta-atom at 0.137 THz. When the right-side VO_2_ patch is heated, the metasurface responds to the normal incidence of (**a**) LCP and (**b**) RCP waves. When the left-side VO_2_ patch is heated, the metasurface interacts to normal incidence of (**c**) LCP and (**d**) RCP waves.

In order to further study the chiral effect of the MIM structure, the electric field distribution of chiral atoms in Figure 3a,b was studied under the normal incidence of CP waves. Figure 5 illustrates the electric field distribution of the two chiral meta-atoms at the resonant frequency 0.137 THz. Figure 5a,b depicts the chiral meta-atoms in the heated state, as shown in Figure 3a, wherein the MIM structure produces a macroscopic vertical electric field under normal incidence of LCP. This electric field subsequently creates a magnetic vortex at the interface between the VO_2_ and Al layers, thereby achieving the spatial localization effect for the LCP. However, this effect is not observed under normal incidence of RCP waves. This difference leads to the emergence of chirality. The other opposite heating state in Figure 5c,d produces a similar electric field distribution when subjected to the incidence of opposing chiral CP waves.

### 2.2. Phase Gradient Configuration

**Figure 6:**
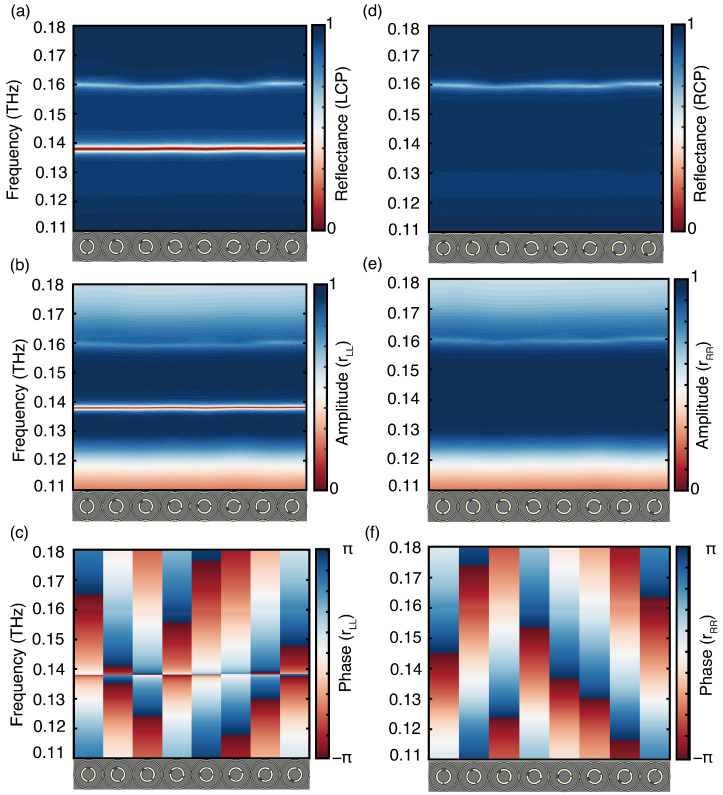
The electromagnetic characteristics of the gradient metasurface composed of eight chiral meta-atoms arranged based on the Pancharatnam–Berry phase theory: (**a**–**c**) show simulation results for (**a**) reflection spectra, (**b**) co-polarized reflection spectra, and (**c**) phase of the chiral meta-atoms when illuminated with LCP wave, while (**d**–**f**) show simulation results for (**d**) reflection spectra, (**e**) co-polarized reflection spectra, and (**f**) phase of the chiral meta-atoms under RCP illumination.

The additional phase shift introduced by the geometric phase metasurface is solely determined by the rotation angle of the meta-atom, which follows a general relationship of twice the rotation angle [47]. This phase manipulation method exhibits wide bandwidth characteristics. To illustrate the metasurface’s abilities in spin-selective absorption and chirality-preserving anomalous reflection, eight meta-atoms were chosen for simulation verification, each with a rotation angle ϕ ranging from 0° to 157.5° in increments of 22.5°. The corresponding electromagnetic responses of LCP and RCP incident waves are shown in Figure 6. Figure 6a,d depicts the variations in reflection spectrum corresponding to LCP and RCP incident waves, respectively, across the eight meta-atoms. Figure 6b,e indicates that the co-polarized reflection amplitudes of these eight meta-atoms remains relatively consistent within the frequency range of 0.13 THz–0.15 THz, with a notably high reflection efficiency (>0.9) at the non-resonant frequency. The corresponding co-polarization reflection phases are shown in Figure 6c,f. At non-resonant frequencies, both the reflected LCP and RCP waves undergo a linear phase change that is twice the rotation angle. This phase change is devoid of dispersion, covering nearly 2π for both LCP and RCP waves. Notably, Figure 6c displays an abrupt resonate change at 0.137 THz, corresponding to Figure 3e. This phenomenon primarily occurs because the chiral meta-atoms efficiently absorb the LCP wave at the resonant frequency while simultaneously reflecting the RCP wave.

### 2.3. Spin-Selective Anomalous Reflection

The eight chiral meta-atoms are integrated into a supercell to establish a gradient phase distribution along the *x*-direction. This metasurface, characterized by a gradient phase, alters the direction of reflection for a normally incident electromagnetic wave, effectively achieving anomalous reflection. The reflection angle $\theta_{r}$ of anomalous reflection can be determined using the generalized Snell’s law [48]: $\sin\theta_{r}=\sin\theta_{i}+\lambda_{0}/l$, where $\theta_{i}$ is the incident angle, $\lambda_{0}$ denotes the working wavelength, and *l* is the periodic length of the supercell. The incident angle $\theta_{i}$ is fixed at 0° and the supercell periodic length *l* is 4600 μm. Then, the reflection angle $\theta_{r}$ can be calculated as follows: $\theta_{r}=\pm \sin^{-1}\left(\lambda_{0}/4600\right)$, where the positive sign (+) corresponds to LCP incident waves and the negative sign (−) corresponds with RCP incident waves. At non-resonant frequencies, the chiral metasurface displays identical reflectivity for both LCP and RCP waves. At the resonant frequency of 0.137 THz, it reflects RCP waves at an angle of −28.4° while simultaneously absorbing LCP waves.

**Figure 7:**
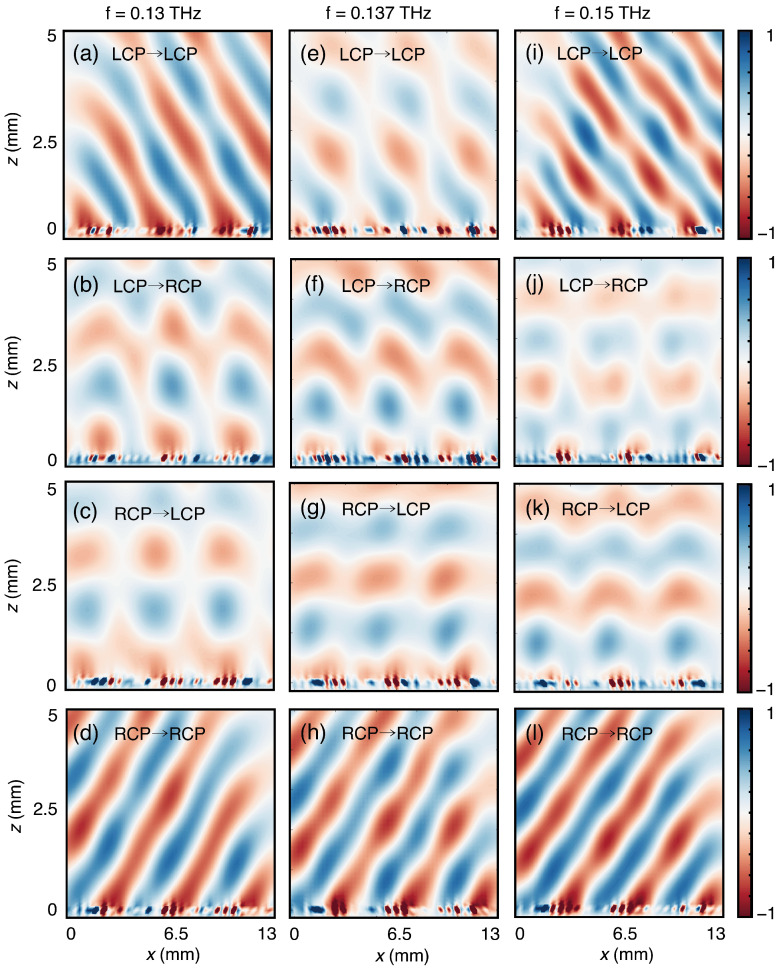
The scattered electric field distributions of the gradient chiral metasurface under normal incidence of different CP waves. The figures on the left (**a**–**d**), middle (**e**–**h**), and right (**i**–**l**) correspond to 0.13 THz, 0.137 THz, and 0.15 THz, respectively.

**Figure 8:**
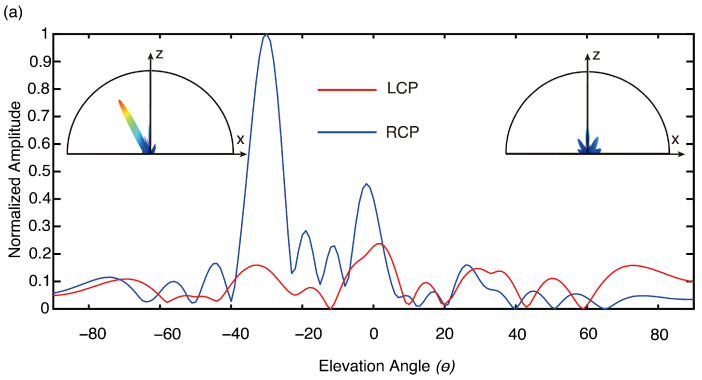

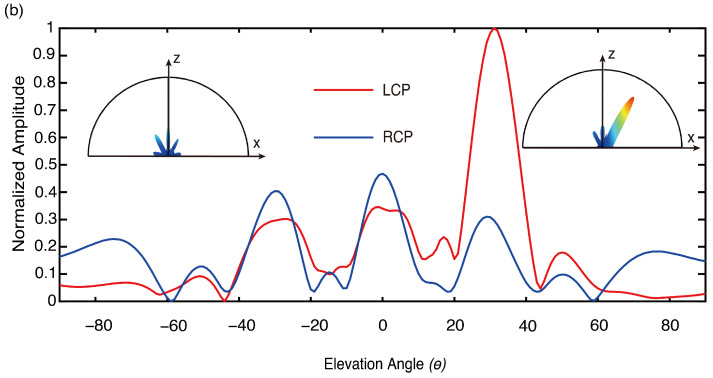
The far-field radiation patterns of the gradient chiral metasurface as a function of the elevation angle when the (**a**) left and (**b**) right VO_2_ patches of the meta-atom are heated into metallic states. The inset images show the 3D far-field radiation patterns for the CP incident waves.

A full wave simulation was performed in order to investigate the scattering performance of the gradient chiral metasurface. Figure 7 illustrates the scattered electric field distributions for CP waves under normal incidences. The CP waves are efficiently deflected at non-resonant frequencies within 0.13 THz–0.15 THz, and polarization conversion is effectively suppressed. At the chiral resonant frequency of 0.137 THz, LCP incident waves are significantly absorbed, and only RCP waves are deflected from the metasurface. The numerical far-field radiation pattern of the metasurface at the resonant frequency 0.137 THz when the metasurface exhibits a chiral structure is shown in Figure 8. The one-dimensional normalized far-field scattering patterns of the metasurface with the left VO_2_ patches in the metallic states, corresponding to the heating state in Figure 3a, are shown in Figure 8a. The inset figures show the three-dimensional (3D) far-field scattering patterns under normal incidences of CP waves. At the chiral resonant frequency of 0.137 THz, LCP waves are significantly absorbed, and only RCP waves are deflected from the metasurface. The one-dimensional far-field normalization results and 3D far-field scattering patterns corresponding to the other opposite heating state in Figure 3b are shown in Figure 8b. While the electromagnetic response is similar, the handedness of the absorbed and reflected waves is reversed. These results show the spin-selective anomalous reflection, polarization selection, and chirality-switching functions of the designed metasurface. By manipulating VO_2_, the metasurface regulates both chiral resonance and polarization channels, effectively controlling the amplitude, phase, and polarization state of CP waves.

## 3. Discussion

In conclusion, this paper proposes a chirality-switchable THz metasurface with integrated heating control circuits, composed of a meta-atom utilizing VO_2_ patches as the switchable component. Employing transmission matrix theory and the principle of PB geometric phase, the metasurface achieves spin-selective anomalous reflection. By driving bias currents to the heating control circuits, the VO_2_ patch can be heated in different states. At non-resonant frequencies within 0.13 THz–0.15 THz, the metasurface suppresses cross-polarization reflection to below 0.2 under CP illumination while simultaneously achieving co-polarization reflection with an efficiency above 0.9. At the resonant frequency of 0.137 THz, it absorbs a specific CP wave while deflecting another, contingent upon the states of the VO_2_ patches. Through a proof-of-concept demonstration, we designed a chiral metasurface featuring a phase gradient arrangement and analyzed the mechanism of spin selection. The switchable chiral metasurface enables spin-selective anomalous reflection of CP waves without altering their handedness at desired angles. Compared to existing metasurfaces, the proposed metasurface exhibits a significant CD effect ranging from −0.938 to 0.938, and possesses the capability to perform diverse functions such as spin-selective absorption and anomalous reflection. With advancements in manufacturing technology, this switchable chiral metasurface holds promise for applications in biomolecular sensing, polarization conversion, 6G encrypted communication, and enlarging wireless communication data capacity.

## Figures and Tables

**Table 1:** A summary of the functionalities of the designed metasurface under various heating conditions.

VO_2_ Heating State	Beam Regulation at the Resonant Frequency	Beam Regulation at Non-Resonant Frequencies
Left: metallic Right: insulated	LCP: Absorbing RCP: Reflecting	LCP: Reflecting RCP: Reflecting
Left: insulated Right: metallic	LCP: Reflecting RCP: Absorbing	LCP: Reflecting RCP: Reflecting
Left: insulated Right: insulated	LCP: Reflecting 50% RCP: Reflecting 50%	LCP: Reflecting RCP: Reflecting
Left: metallic Right: metallic	LCP: Reflecting RCP: Reflecting	LCP: Reflecting RCP: Reflecting

## Data Availability

The data analyzed during the current study are available from the corresponding author upon reasonable request.
